# Silent cerebral infarcts in patients with sickle cell disease: a systematic review and meta-analysis

**DOI:** 10.1186/s12916-020-01864-8

**Published:** 2020-12-22

**Authors:** Maite E. Houwing, Rowena L. Grohssteiner, Marjolein H. G. Dremmen, Ferdows Atiq, Wichor M. Bramer, Anne P. J. de Pagter, C. Michel Zwaan, Tonya J. H. White, Meike W. Vernooij, Marjon H. Cnossen

**Affiliations:** 1 Department of Pediatric Haematology and Oncology, Erasmus MC – Sophia Children’s Hospital, NC-825, Wytemaweg 80, 3015 CN Rotterdam, The Netherlands; 2 Department of Pediatric Radiology, Erasmus MC – Sophia Children’s Hospital, Rotterdam, The Netherlands; 3 Department of Haematology, Erasmus MC, Rotterdam, The Netherlands; 4 Medical Library, Erasmus MC, Rotterdam, The Netherlands; 5 Princess Máxima Center for Pediatric Oncology, Utrecht, The Netherlands; 6 Department of Child and Adolescent Psychiatry, Erasmus MC – Sophia Children’s Hospital, Rotterdam, The Netherlands; 7 Department of Radiology and Nuclear Medicine, Erasmus MC, Rotterdam, The Netherlands; 8 Department of Epidemiology, Erasmus MC, Rotterdam, The Netherlands

**Keywords:** Sickle cell disease, Silent cerebral infarction, Stroke, Magnetic resonance imaging

## Abstract

**Background and purpose:**

Silent cerebral infarcts (SCIs) are the most common neurological complication in children and adults with sickle cell disease (SCD). In this systematic review, we provide an overview of studies that have detected SCIs in patients with SCD by cerebral magnetic resonance imaging (MRI). We focus on the frequency of SCIs, the risk factors involved in their development and their clinical consequences.

**Methods:**

The databases of Embase, MEDLINE ALL via Ovid, Web of Science Core Collection, Cochrane Central Register of Trials via Wiley and Google Scholar were searched from inception to June 1, 2019.

**Results:**

The search yielded 651 results of which 69 studies met the eligibility criteria. The prevalence of SCIs in patients with SCD ranges from 5.6 to 80.6% with most studies reported in the 20 to 50% range. The pooled prevalence of SCIs in HbSS and HbSβ^0^ SCD patients is 29.5%. SCIs occur more often in patients with the HbSS and HbSβ^0^ genotype in comparison with other SCD genotypes, as SCIs are found in 9.2% of HbSC and HbSβ^+^ patients. Control subjects showed a mean pooled prevalence of SCIs of 9.8%. Data from included studies showed a statistically significant association between increasing mean age of the study population and mean SCI prevalence. Thirty-three studies examined the risk factors for SCIs. The majority of the risk factors show no clear association with prevalence, since more or less equal numbers of studies give evidence for and against the causal association.

**Conclusions:**

This systematic review and meta-analysis shows SCIs are common in patients with SCD. No clear risk factors for their development were identified. Larger, prospective and controlled clinical, neuropsychological and neuroimaging studies are needed to understand how SCD and SCIs affect cognition.

**Supplementary Information:**

The online version contains supplementary material available at 10.1186/s12916-020-01864-8.

## Background

Sickle cell disease (SCD) is an autosomal recessive haemoglobinopathy characterized by ongoing haemolytic anaemia, episodes of vaso-occlusion and progressive organ failure. Millions are affected worldwide, and approximately 312.000 neonates with this haematological disorder are born annually [1]. SCD is caused by a single nucleotide substitution in codon 6 of the β-globin gene. This mutation leads to the formation of abnormal haemoglobin, called HbS [2]. When deoxygenated, HbS erythrocytes become sickle or crescent-shaped, rigid and prone to lysis. These sickle cells interact with leukocytes and the vascular endothelium causing occlusion and vasculopathy, subsequently leading to a broad range of acute and chronic complications including cerebrovascular disease [3, 4].

The most common neurological complication in children and adults with SCD is the development of silent cerebral infarcts (SCIs), also referred to as silent strokes [5–7]. In contrast to the clinically overt strokes, SCIs do not lead to apparent focal neurological symptoms and can only be detected with neuroimaging techniques [8, 9]. As a consequence, SCIs are identified incidentally or through screening. Although SCIs do not lead to any tangible motor or sensory deficits, they are associated with cognitive morbidity and an increased risk of future strokes [10–12]. SCIs are visible as focal lesions on both computed tomography (CT) scans and magnetic resonance imaging (MRI) scans. Detection is however better by MRI due to the greater range of contrast between soft tissues and greater detail in the depiction of intracranial structures [13].

There is an ongoing debate over the rationale of screening for SCIs in patients with SCD [14, 15]. While the Silent Cerebral Infarct Transfusion (SIT) randomized controlled trial showed that chronic red blood cell transfusions reduce the risk of recurrent infarction, this benefit was incomplete with some children in the transfusion therapy arm also developing infarct recurrence [16]. More importantly, the true incidence and prevalence of SCIs remain unknown and the understanding of the pathophysiology and risk factors limited. In this systematic review, we provide an overview of studies that have used brain MRI studies to detect SCIs in patients with SCD while focusing on the frequency of SCIs, the risk factors potentially involved in their occurrence and their clinical impact. Emphasis is placed on the epidemiology of SCIs and not on the evaluation of intervention studies.

## Methods

### Article retrieval

For this report, the Preferred Reporting Items for Systematic Reviews and Meta-Analyses (PRISMA) guidelines were followed [17]. A comprehensive systematic search was performed in Embase, MEDLINE ALL via Ovid, Web of Science Core Collection, Cochrane Central Register of Trials via Wiley and Google Scholar (Additional file 1) from inception to June 1, 2019. Search terms included multiple synonyms for ‘SCIs’, ‘SCD’ and ‘MRI’ in various combinations. No limitations in the search strategy were inserted. The search strategy was designed and conducted by an experienced librarian (W.M.B.) with input from the primary investigators.

### Study selection

Studies were screened on potential eligibility by two independent reviewers (M.E.H., R.L.G.). Papers had to be written in English. Studies had to report original data; reviews, case reports and letters were excluded. Both controlled studies and (retrospective) cohort studies were eligible. Studies were included if they involved patients of all ages with either homozygous or compound heterozygous SCD without overt stroke and specifically assessed for detection of SCIs by MRI. Differences of opinion were resolved by discussion and consensus between the reviewers.

### Data assessment

For each included study, the following information was collected: study design, characteristics of the patient population, mean or median age at study inclusion, utilized MRI protocol, additionally performed advanced MR and ultrasonic imaging techniques, SCI prevalence, risk factors studied, clinical consequences and other major observations or conclusions. For any missing information or unresolved discrepancies, we contacted the authors of the studies for clarification or to request unpublished data. As it is difficult to determine whether a focal hyperintensity seen on MRI is caused by actual infarction or by another underlying cause (e.g. inflammation, infection, demyelination) [18], all different terms used for strongly related MRI findings in included studies (e.g. white matter hyperintensities, white matter changes, silent lesions, ischaemic lesions) were considered to be SCIs. The reviewers read and abstracted each article, and a third member with specific imaging expertise (M.H.D) checked the table entries for accuracy with regard to the original articles. Data were reviewed descriptively.

Where multiple articles were included for a single or overlapping population sample, prevalence and incidence estimates were obtained from the report with the largest sample size to prevent duplication.

### Statistical methods

Continuous data are presented as mean and range or as 95% confidence intervals (CI), whereas categorical data are presented as frequency and proportion (%). The mean pooled prevalence of SCIs was calculated with univariate general linear models, in which we weighted SCI prevalence for the sample size of the included studies. The outcomes are presented as pooled mean prevalence and 95% CI. We also used univariate general linear models to compare the mean pooled prevalence of SCIs between the two independent groups.

The association between the prevalence of SCIs and age was analysed with linear regression analysis. We adjusted the outcomes of the linear regression analysis for publication year, study design (i.e. prospective cohort study or retrospective cohort study), sample size and field strength. The outcomes of the linear regression analysis are presented as unstandardized beta (*β*), 95% CI and *p* value.

For studies that did not report the mean age of study participants, grouped data mean calculation formula was used to calculate the mean. For open-end age intervals (e.g. < 25 or > 50 years) where it is not possible to calculate the mean, the median and calculated median of study participants were applied.

### Risk factor analysis

Selected articles were evaluated to identify all studied risk factors. The reported statistics described in univariate analyses were examined to determine the direction of the association of a particular risk factor and whether it was deemed statistically significant. ‘Independent’ risk factors were identified from studies in which multivariable analyses were conducted.

## Results

**Fig. 1:**
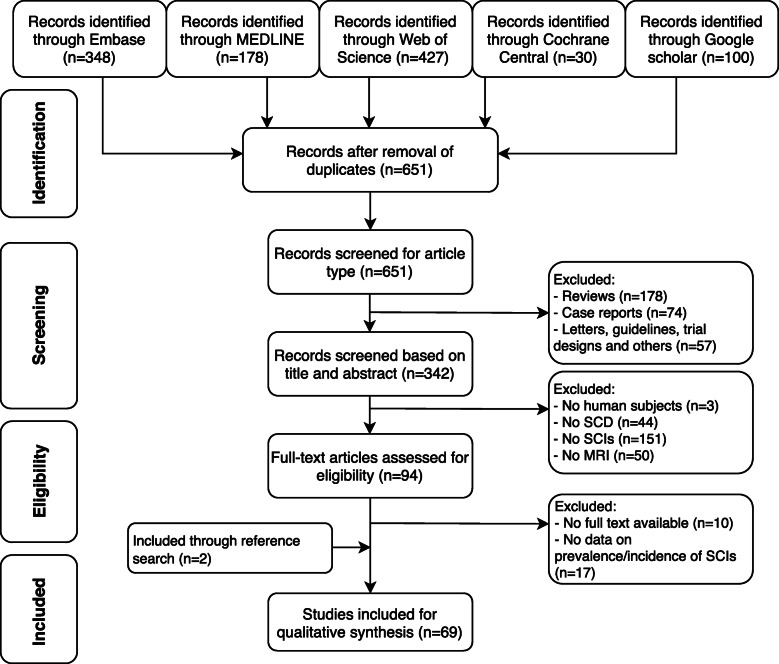
Flowchart of publications included in the systematic review. SCD, sickle cell disease; SCIs, silent cerebral infarcts; MRI, magnetic resonance imaging

The literature search yielded a total of 651 non-duplicate citations that were screened using predetermined inclusion and exclusion criteria. A total of 69 full-text articles met the inclusion criteria for this review. These publications reported data on the frequency of SCIs in patients with SCD from 41 studies. Figure 1 shows the flow chart of articles resulting from the initial search to the final inclusion or exclusion. Twenty-three studies were conducted in the USA (58.5%), 14 in Europe, one in Brazil, one in India, one in Turkey and one in Kuwait.

### Definitions of SCIs and MRI detection

An important finding was the general lack of uniformity in definitions of SCIs in SCD. Definitions identified were based on both MRI criteria and clinical characteristics (e.g. normal neurological examination). In most studies, SCIs were defined as focal areas of abnormally increased T2-weighted signal intensity on multiple anatomical views without associated neurological deficits. Approximately half of the studies applied a more precise definition in which a focal brain lesion was required to be at least 3 or 5 mm in one dimension and visible in two planes on fluid-attenuated inversion recovery (FLAIR) T2-weighted images, with a normal neurological examination. Eight studies did not explicitly state what the applied definition for SCIs was.

In addition to the varying definitions of SCIs, the detection of SCIs depends on MRI parameters including magnet strength and spatial resolution (most importantly slice thickness). The majority of studies (72%) used a 1.5-T MRI magnet, a 3.0-T magnet was used in 17% of studies and the remaining 11% used another magnet strength (i.e. 0.6 T, 1.0 T, 7.0 T). Slice thickness varied from 1.0 to 6.0 mm and was not mentioned in 24 (58.5%) studies.

Information concerning MRI parameters—including magnet strength and sequences—as well as imaging and clinical criteria used for diagnosis of SCIs, in the 41 included studies, is depicted in Table 1.

**Table 1:** Applied definitions and magnetic resonance imaging parameters in studies of silent cerebral infarcts in sickle cell disease patients

Author (year)	Magnet strength	Slice thickness (mm)	Sequence	MRI criteria for SCIs detection	Clinical criteria for SCIs
Abboud et al. 2011 [19]	–	5.0	T1: ax | FLAIR: ax, cor	Evidence of cerebral infarction on MRI	No history of overt stroke
Arkuszewski et al. 2014 [20]	3.0 T	–	FLAIR	Area of abnormal hyperintensity, ≥ 3 mm on FLAIR, visible on at least two perpendicular planes	–
Asbeutah et al. 2014 [21]	1.5 T	5.0	T1: ax, sag | T2: ax, sag | FLAIR	–	–
Baldeweg et al. 2006 [22]	1.5 T	–	T1: sag, cor | T2: ax | FLAIR: cor	Area of abnormal hyperintensity on T2	No history of a focal neurological deficit lasting > 24 h
Bernaudin et al. 2011 [23]	1.0 or 1.5 T	–	T1 | T2 | FLAIR	Signal abnormality, ≥ 3 mm in one dimension, visible on two views on T2	No history of overt stroke and normal neurologic examination
De Blank et al. 2010 [24]	–	–	–	Evidence of cerebral infarction on MRI	No overt neurological symptoms
Brousse et al. 2017 [25]	–	–	–	–	–
Brown et al. 2000 [26]	–	–	–	Evidence of cerebral infarction on MRI	No history of overt stroke and normal neurological examination
Calvet et al. 2017 [27]	3.0 T	–	T2 | FLAIR	White matter lesions, i.e. poorly defined hyperintensities, ≥ 5 mm on T2 or FLAIR	No history of overt stroke or overt neurological symptoms
Coloigner et al. 2017 [28]	3.0 T	1.0 or 1.3 or 5.0	3D T1 | 3D T2 |	≥ 3 mm lesions on 3D T2 observed in two orthogonal planes	No history of overt stroke
Dowling et al. 2012 [29]	1.5 or 3.0 T	–	FLAIR: ax	Area of abnormal hyperintensity intensity in multiple T2	No history or physical findings of a focal neurological deficit lasting > 24 h
Ford et al. 2017 [30]	–	–	T1 |FLAIR	Area of abnormal hyperintensity on FLAIR > 3 mm and cerebrospinal fluid-like hypointensity on T1	–
Ford et al. 2018 [31]	–	–	T1 |FLAIR	Signal abnormality, ≥ 3 mm in one dimension, visible on two planes on FLAIR T2	Normal neurological examination or absence of neurological symptoms that correlate with lesion location
Gold et al. 2008 [32]	–	–	–	–	–
Guilliams et al. 2015 [33]	1.5 or 3.0 T	5.0	T1: sag | T2: ax |FLAIR: ax, cor	Signal abnormality, ≥ 3 mm in one dimension, visible on two planes on T2 or FLAIR	Absence of neurological symptoms that correlate with lesion location
Gyang et al. 2011 [34]	–	–	–	Abnormal MRI changes	No neurological symptoms
Issar et al. 2018 [35]	1.5 T	–	T1: ax | T1 FLAIR: sag | T2: ax |FLAIR: ax	Areas of abnormal hyperintensity on FLAIR T2	Absence of overt clinical neurological symptoms
Kassim et al. 2016 [36]	–	–	T2	≥ 3 mm on T2 in two imaging planes	No history of neurological deficits and normal neurological examination
Kawadler et al. 2018 [37]	1.5 T	–	T2: ax	–	–
Kwiatkowski et al. 2009 [6]	1.5 T	–	FLAIR	Area of abnormal hyperintensity on T2 and FLAIR	No history of overt stroke or motor deficits that can be attributed to the lesion
Melek et al. 2006 [38]	1.5 T	5.0	T1: sag, ax | T2: sag, ax | PD: ax	Abnormal MRI	No history or physical findings of a focal neurological deficit lasting > 24 h
Mercuri et al. 1995 [39]	1.0 T	6.0	T2	–	–
Oguz et al. 2003 [40]	1.5 T	5.0	T1: sag | T2: ax | FLAIR	–	–
Onofri et al. 2012 [41]	1.5 T	5.0	FLAIR: ax	–	–
Pegelow et al. 2001 [42]	–	5.0	T1: ax | PD T2: ax, cor	–	–
Pegelow et al. 2002 [10]	–	–	–	Abnormal MRI	No neurological deficit
Quinn et al. 2013 [43]	–	–	FLAIR	Signal abnormality ≥ 3 mm in one dimension, visible on at least two views of T2 FLAIR	Normal neurological examination or absence of neurological symptoms that correlate with lesion location
Schatz et al. 2006 [44]	1.5 T	5.0	T1: sag | T2: ax	Area of abnormal hyperintensity, ≥ 3 mm on T2	Normal neurological history
Seibert et al. 1993 [45]	1.5 T	–	T1 | T2 | PD: ax	–	–
Silva et al. 2009 [46]	1.5 T	5.0	T1: sag, ax | T2 | FLAIR: ax	Evidence of ischaemia, including lacunar infarction, encephalomalacia, atrophy or leukoencephalopathy	–
Solomou et al. 2013 [47]	1.0 T	5.0	T2: ax, cor | FLAIR: ax, cor	Focal (< 1 cm) or multiple (> 1 cm) high-intensity lesions on T2 or FLAIR	Absence of physical findings of overt stroke
Steen et al. 2003 [48]	1.5 T	5.0 or 3.0	T1: ax | T2: ax |FLAIR: ax	Evidence of ischaemia, including lacunar infarction, encephalomalacia, atrophy or leukoencephalopathy	Absence of a clinical history of stroke
Tewari et al. 2018 [49]	–	–	FLAIR	Area of abnormal hyperintensity ≥ 3 mm in diameter and visible in at least two planes of T2 (ax and cor)	No history or physical findings of a focal neurological deficit in a corresponding localizing vascular distribution
Václavů et al. 2019 [50]	3.0 T	–	3D FLAIR	Multiple (> 1) hyperintensities ≥ 5 mm	–
Van der Land et al. 2015 [51]	3.0 and 7.0 T	–	T1 |FLAIR: ax	Areas of abnormal hyperintensity	No history or physical findings of a focal neurological deficit
Van der Land et al. 2016 [52]	3.0 T	5.0	T2 |FLAIR	Hyperintensity of variable size in the white matter on FLAIR, without cavitation	No history or physical findings of a focal neurological deficit
Vichinsky et al. 2010 [53]	1.5 T	–	T1 | T2 | PD	Area of abnormal hyperintensity at least 5 mm on T2 and PD, with corresponding hypointensity on T1	–
Wang et al. 1998 [54]	1.5 T	5.0	T1: ax | T2: ax	Area of abnormal hyperintensity on T2	No history of neurological symptoms compatible with overt stroke
Wang et al. 2008 [55]	1.5 T	5.0	T1: ax |FLAIR: ax, cor	Area of abnormal hyperintensity on T2, consistent with an ischaemic lesion in white matter	–
Watkins et al. 1998 [56]	1.5 T	5.0	T2: ax	–	–
Zafeiriou et al. 2004 [57]	1.5 T		T1: ax	Area of abnormal hyperintensity on T2	–

*MRI* magnetic resonance imaging, *SCIs* silent cerebral infarctions, *ax* axial, *cor* coronal, *sag* sagittal, *T1* T1-weighted, *T2* T2-weighted, *FLAIR* fluid-attenuated inversion recovery, *PD* proton density

### Additional neuroimaging techniques

#### Transcranial Doppler

The majority of studies excluded patients with abnormal transcranial Doppler (TCD) flow velocities (> 170 cm/s) indicative of an increased risk of overt stroke. Most studies that concomitantly measured TCD velocities found no significant differences between the mean TCD velocities of patients with normal MRI scans and patients with detected SCIs [6, 23, 57–64]. Moreover, comparison of patients who were included in both the Cooperative Study of SCD (CSSCD) and the Stroke Prevention (STOP) trial showed that patients with abnormal TCDs did not demonstrate a concurrent high prevalence of SCIs. Conversely, those who had SCIs did not present with a high prevalence of abnormally increased TCD velocities [64].

In contrast, two studies—one retrospective cohort study and one prospective cohort study—in respectively 254 and 23 SCD patients reported a significant association between higher (maximum) TCD velocities and SCIs [24, 55].

#### Magnetic resonance angiography

More than half of the included studies performed magnetic resonance angiography (MRA) as part of the MRI examination. Some studies found that signs of MRA-defined cerebral vasculopathy were related to the presence and/or number of SCIs [6, 20, 24, 58, 63]. In the Silent Cerebral Infarct Transfusion (SIT) trial, the frequency of intracranial vasculopathy in patients with and without SCIs was 15.9% and 6.3%, respectively (*p* < 0.001). However, the majority (84%) of patients with SCIs did not show vasculopathy on MRA [63].

#### Arterial spin labelling

Arterial spin labelling (ASL) provides a method to non-invasively obtain a quantitative measurement of cerebral blood flow. The majority of recently performed ASL studies confirm an elevated global cerebral blood flow in patients with SCD, with no differences between patients with and without SCIs [40, 52, 65, 66]. However, a study by Ford et al. compared cerebral blood flow maps from children with and without SCIs and found that SCIs were associated with impaired haemodynamics including low cerebral blood flow in the region of the highest infarct density (*p* < 0.001) [31].

### SCI localization

Approximately 80% of children in whom SCIs were detected in the CSSCD study had abnormalities in the frontoparietal deep white matter and periventricular regions on MRI, with other infarcts located in the basal ganglia and the temporal lobe. Infarcts were distributed equally in both brain hemispheres [8, 10]. Similar observations were reported in other studies [20, 22, 28, 30, 33, 50, 55, 59, 62]. Both overt strokes and SCIs predominantly occurred in the watershed regions of the deep white matter and encompassed only 5.6% of the brain volume [31].

### SCI frequency

The prevalence and incidence of SCIs in patients with SCD varied widely, depending on the study population and MRI protocol. While most included studies were cohort studies, both prospective and retrospective, some case-control studies and one randomized controlled trial were also included. We examined both prevalence (i.e. proportion of patients with SCIs at one particular time) and incidence (i.e. rate at which patients develop SCIs over time). One study in 23 individuals found a SCI prevalence rate that extremely deviated from the prevalences observed in other included studies, with a *Z*-score of 2.7 [51]. The prevalence of SCIs was most probably much higher in this study, due to the use of a 7-T MRI scan. To not distort the results, this study was deemed an outlier and excluded from frequency analyses.

#### Prevalence of SCIs in HbSS and HbSβ^0^ genotypes

Most studies (*n* = 27) were performed in patients with HbSS and HbSβ^0^ SCD (*n* = 2789), with overall prevalence rates ranging from 5.6 to 80.6% (Table 2). The pooled prevalence of SCIs in HbSS and HbSβ^0^ genotypes was 29.5% (95% CI 25.2–33.9).

**Table 2:** Prevalence of silent cerebral infarcts in HbSS and HbSβ^0^ sickle cell disease patients

Author (year)	Study design	Sample size	Mean age (years)	Field strength (T)	Prevalence SCIs (%)
Abboud et al. 2011 [19]	PCS	77	12.3	NA	26.6
Arkuszewski et al. 2014 [20]	PCS	67	8.8	3.0	37.7
Asbeutah et al. 2014 [21]	PCS	40	10.1*	1.5	10
de Blank et al. 2010 [24]	RCS	254	10.6	NA	30.7
Brousse et al. 2017 [25]	PCS	59	11.4	NA	13.6
Brown et al. 2000 [26]	PCS	48	9.8*	NA	22.9
Calvet et al. 2017 [27]	RCS	83	43.3	3.0	49.4
Ford et al. 2017 [30]	PCS	22	27	NA	54.5
Ford et al. 2018 [31]	PCS	1061	NA	NA	27
Guilliams et al. 2015 [33]	RCS	168	NA	1.5/3.0	27.4
Gyang et al. 2011 [34]	RCS	8	15	NA	50
Kassim et al. 2016 [36]	RCS	60	30	NA	53.3
Kwiatkowski et al. 2009 [6]	RCS	65	3.6	1.5	27.7
Marouf et al. 2003 [67]	PCS	35	26.9	NA	20
Mercuri et al. 1995 [39]	PCS	11	9.3	1.0	45.5
Nottage et al. 2016 [68]	PCS	50	9.4	NA	38
Oguz et al. 2003 [40]	PCS	18	8.7	1.5	5.6
Pegelow et al. 2002 [10]	RCS	266	8.3*	NA	21.8
Schatz et al. 2005 [44]	PCS	20	12.2**	1.5	40
Silva et al. 2009 [46]	PCS	46	26.8	1.5	56.5
Tewari et al. 2018 [49]	PCS	51	12.4**	NA	37.3
Václavů et al. 2019 [50]	PCS	36	31.9	3.0	80.6
Van der Land et al. 2015 [51]	PCS	10	23	3.0/7.0	50
Van der Land et al. 2016 [52]	PCS	34	12.1	3.0	41.2
Vichinsky et al. 2010 [53]	PCS	141	31.6	1.5	28.9
Wang et al. 1998 [54]	PCS	36	1.5	1.5	8.3
Wang et al. 2008 [55]	PCS	23	1.1	1.5	13

*SCIs* silent cerebral infarctions, *PCS* prospective cohort study (including case-control and randomized controlled trial), *RCS* retrospective cohort study, *NA* not available

*Only the mean age for the full sample was provided, not separately for HbSS and HbSβ^0^ thalassemia patients

**Only the mean age for the SCI-affected and SCI-unaffected group separately was provided, not for the full sample

**Fig. 2:**
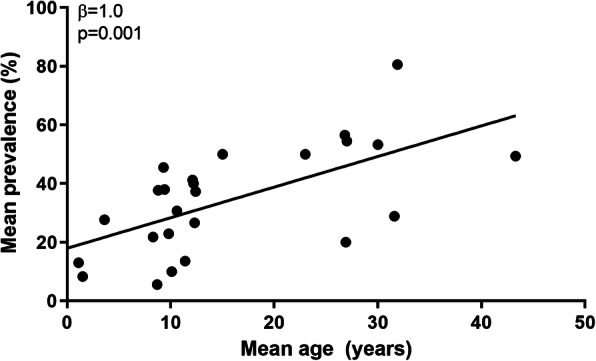
The mean prevalence of silent cerebral infarcts, by mean age in patients with HbSS and HbSβ^0^ sickle cell disease. Linear regression analysis was used to analyse the association between age and SCI prevalence

Data from the included studies showed a statistically significant association between increasing mean age of the study population and mean SCI prevalence: *β* = 1.0% increase in SCI prevalence for 1 year increase in mean age (95% CI 0.5–1.6), *p* = 0.001, Fig. 2. When corrected for publication year, study design (i.e. prospective cohort study or retrospective cohort study), sample size and field strength, the association between age and SCI prevalence remained significant: *β* = 0.8% (95% CI 0.2–1.5), *p* = 0.012.

#### Prevalence of SCIs in other genotypes and healthy controls

**Fig. 3:**
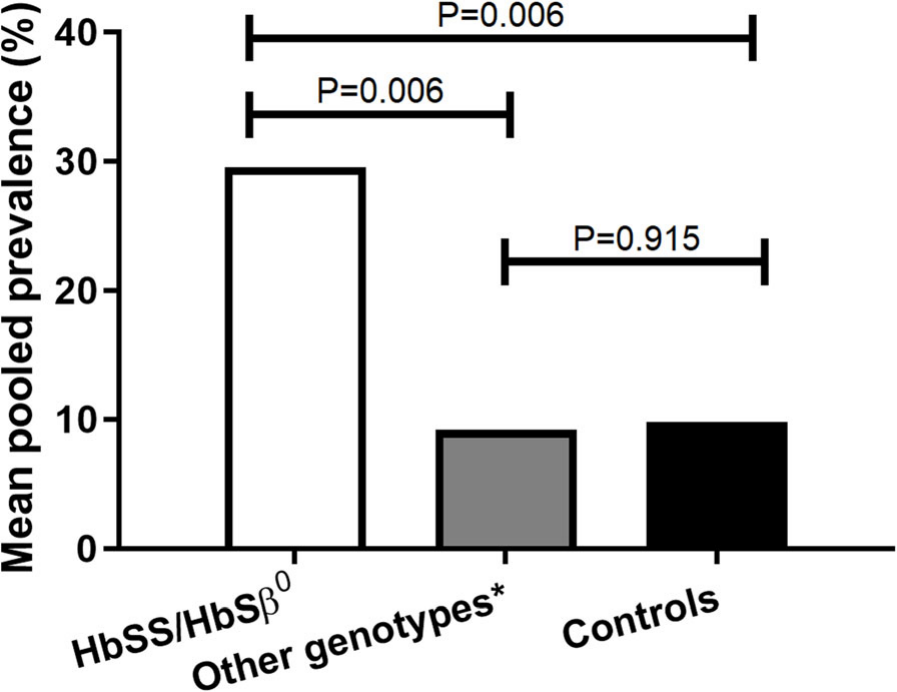
Comparison of the mean prevalence of silent cerebral infarcts in different sickle cell disease genotypes versus healthy controls. Univariate general linear models were used to compare the mean pooled prevalence of SCIs between the two groups.*HbSC, HbSβ^+^, HbSE

Twelve studies separately reported the prevalence of SCIs in other SCD genotypes, i.e. HbSC, HbSβ^+^ (total *n* = 254), and in healthy controls (*n* = 266) [10, 21, 22, 26, 28–30, 33, 35, 40, 50, 53]. The mean pooled prevalence of SCIs was 9.2% (95% CI 2.9–15.4) in patients with HbSC and HbSβ^+^, which was significantly lower compared to patients with HbSS and HbSβ^0^ SCD (*p* = 0.006, Fig. 3).

In nine studies (total *n* = 266), the prevalence of SCIs was reported for controls [21, 22, 28–30, 35, 40, 50, 53]. Control subjects were mostly matched adolescents or young adult family members, with a mean age varying between 9.8 and 37.4 years. Although healthy, information regarding possible HbS carrier status was not provided for all controls. Sickle cell trait (HbAS genotype) was reported in 40 control subjects, whereas 132 subjects were explicitly reported not to have sickle cell trait (i.e. HbAA). Carrier status was unknown for the remaining 94 controls. Additional Table 1 shows the prevalence of SCIs and information regarding carrier status for control subjects. Control subjects showed a mean pooled prevalence of SCIs of 9.8% (95% CI − 0.5–20.1). Surprisingly, there was no significant difference (*p* = 0.915) in the mean pooled prevalence of SCIs between patients with HbSC and HbSβ^+^ SCD and controls (Fig. 3).

#### SCI incidence

Incidence data in different age groups was scarce, due to the inherent difficulties of longitudinal studies. Only four studies provided estimates ranging from 3.1 to 13.6% per year [10, 23, 34, 43]. In addition, the majority of studies did not address the first presentation of SCIs in young children, as most studies included patients older than 6 years of age because younger children often need sedation during MRI studies. However, four studies did report results from very young children, showing that SCIs begin to develop in children as young as 1 year of age [6, 54, 55, 59].

### Risk factors

Thirty-three studies were identified that examined the risk factors for SCIs in patients with SCD (Table 3). Remarkably, for most risk factors, equal numbers of studies were found showing significant as well as insignificant associations. Furthermore, conflicting results were founded for two risk factors, i.e. mean systolic blood pressure and MCV with studies finding positive associations and studies finding negative associations.

**Table 3:** Summary of the risk factors for MRI-defined silent cerebral infarcts in sickle cell disease

	Abboud et al. 2011* [19]	Arkuszewski et al. 2014 [20]	Armstrong et al. 1996** [12]	Asbeutah et al. 2014^○^ [21]	Baldeweg et al. 2006 [22]	Bernaudin et al. 2000^ǂ^ [69]	Bernaudin et al. 2011^ǂ^ [23]	Bernaudin et al. 2015^ǂ^ [58]	de Blank et al. 2010 [24]	Brousse et al. 2017 [25]	Calvet et al. 2017 [27]	DeBaun et al. 2012 [70]	Farina et al. 2012 [60]	Jordan, Kassim et al. 2018 [71]	Jordan, Williams et al. 2018 *** [61]	Kinney et al. 1999 [72]	Kwiatkoswki et al. 2009 [6]	Marouf et al. 2003 ○ [67]	Melek et al. 2006 [38]	Miller et al. 2001** [11]	Nottage et al. 2016 [68]	Ogunsile et al. 2018*** [73]	Pegelow et al. 2001* [42]	Pegelow et al. 2002** [10]	Sarnaik et al. 2009*** [74]	Silva et al. 2009 [46]	Solomou et al. 2013 [47]	Strouse et al. 2009 [75]	Tewari et al. 2018 [49]	Van der Land et al. 2016 [52]	Wang et al. 2000*,** [64]	Wang et al. 2008 [55]	Zafeiriou et al. 2004 [57]
Sample size	77	67	194	40	31	133	132	189	254	59	83	814	31	421	54	230	65	35	59	248	50	958	127	415	542	46	24	76	51	34	78	23	21
**Demographic factors**																																	
Age	0	0	0	+	0	+				0	+	0		0	0	0	0	+	+			+	+	+			0		0	0	0	0	0
Male sex	0	0	0	0	0		0	0				+		0	0	0	0							0	+		0		0	+	0		0
**Physical findings**																																	
Mean SBP										−	+	+		0	0	0					0								+	0			
SAO2								0				+									+				0								
BMI														0		0																	
**Laboratory findings**																																	
WBC count	0	0		0			0	0			0	0		0		+		0			0				0			0				0	
ANC														0							0								0				
RBC count		0									0							0															
MCV		0					0	−			−							0			+												
MCH											−							0											0				
PC	0	0		0		+	0	0			0					0		0			0											0	
ARC	0	0					0	0			0			0	+	0		0			0				0			0	0	0			
Ht		0	−	0						−	0																						0
TBIL	0										0					0	0												0				
AST																0													0				
LDH	0			0			0	0			0										0							0	0	0			
Hb	0	0		0			−	−			0	−		0	−	−	−	0			−	−			0			−	0	0	0	0	0
HbF%				0			0	+			+	0				0	0	0			+				+			0	+	0		+	0
HbS%	0																																
Apo A1																												+	+				
Apo B																												0	+				
**Imaging findings**																																	
MTCD				0					+								0														0	0	0
ICS		+						+	+					0			+									+				0			
ECS								+						0												+							
**Medical history**																																	
α-thal	0						0	0		0						0		0												0			
G6PD							0	0		0																			0				
SEN																																	
VOC								0		−		0				−	−								0								
AAE								+								0	0																
ACS								0				0				0	−								0							0	
B19V																						+											
Seizure																+																	0
HA											0	0																					
**Treatment**																																	
HU										0				0	0		0										0		0	0			
CTx	−													0	0		0																

‘+’ indicates a statistical significant positive association between the risk factor associated and SCI prevalence; ‘−’ indicates a statistical significant negative association between the risk factor associated and SCI prevalence; ‘0’ indicates the risk factor had failed to reach statistical significance; an empty cell indicates the risk factor was not studied

*Abbreviations*: *SBP* systolic blood pressure, *SAO*_*2*_ oxygen saturation, *BMI* body mass index, *WBC* white blood cell, *ANC* absolute neutrophil count, *RBC* red blood cell, *MCV* mean corpuscular volume, *MCH* mean corpuscular haemoglobin, *PC* platelet count, *ARC* absolute reticulocyte count, *Ht* haematocrit, *TBIL* total bilirubin, *AST* aspartate aminotransferase, *LDH* lactate dehydrogenase, *Hb* haemoglobin, *HbF* foetal haemoglobin, *Apo A1* apolipoprotein A1, *Apo B* apolipoprotein B, *MTCD* mean transcranial Doppler velocity, *ICS* intracranial stenosis, *ECS* extracranial stenosis, *α-thal* α-thalassemia presence, *G6PD* glucose-6-phosphate dehydrogenase deficiency, *SEN* Senegal β-globin haplotype, *VOC* vaso-occlusive crisis rate, *AAE* acute anaemic event, *ACS* acute chest syndrome rate, *B19V* parvovirus B19 infection, *HA* headaches, *HU* hydroxyurea, *CTx* transfusion

*Stroke Prevention (STOP) trial

**The Cooperative Study of Sickle Cell Disease (CSSCD)

***Silent Cerebral Infarct Transfusion (SIT) trial

^ǂ,○^As these studies took place at identical clinical sites, an overlap in the study population may exist

Seven studies reported nine ‘independent’ risk factors in multivariable analyses (Table 4). Of these risk factors, only systolic blood pressure, haemoglobin level and foetal haemoglobin percentage were shown to be significant in more than one model. It was not possible to estimate a pooled odds ratio (OR) or a mean difference due to the small number of studies available.

**Table 4:** Summary of the risk factors for MRI-defined silent cerebral infarcts in sickle cell disease analysed in multivariate models

Risk factors	OR/HR* (95% CI)	*p* value	Study
Male sex	NA	0.030	DeBaun et al. 2012** [70]
Higher SBP	NA	0.018	DeBaun et al. 2012** [70]
	NA	0.015	Sarnaik et al. 2009 [74]
Higher WBC count	3.23 (1.24–14.37)	0.016	Kinney et al. 1999 [72]
Lower Hb level	1.75 (1.14–2.78)	0.011	Bernaudin et al. 2011 [23]
	2.88 (1.05–7.87)	0.039	Bernaudin et al. 2015 [58]
	NA	0.001	DeBaun et al. 2012** [70]
Lower HbF%	0.84 (0.72–0.97)	0.02	Calvet et al. 2017 [27]
	NA	0.038	Sarnaik et al. 2009 [74]
Apolipoprotein A1	0.96	< 0.05	Strouse et al. 2009 [75]
Extracranial stenosis	3.11 (1.10–8.85)	0.033	Bernaudin et al. 2015 [58]
SEN β-globin haplotype	2.53 (1.03–6.23)	0.044	Kinney et al. 1999 [72]
VOC rate	0.53 (0.30–0.95)	0.034	Kinney et al. 1999 [72]
Acute anaemic event	3.39 (1.01–11.34)	0.048	Bernaudin et al. 2015 [58]
Seizure	14.4 (1.5–141)	0.023	Kinney et al. 1999 [72]

*Abbreviations*: *NA* not available, *SBP* systolic blood pressure, *WBC* white blood cell, *Hb* haemoglobin, *HbF* foetal haemoglobin, *SEN* Senegal, *VOC* vaso-occlusive crisis

*Results reported as odds ratio (OR) or hazard ratio (HR)

**This reduced multivariate logistic regression model was overall statistically significant (*x*^2^ = 24.41, df = 3, *p* = .001)

### Clinical impact

By definition, SCIs do not lead to overt neurological symptoms. However, they are associated with more subtle neurological deficits and an increased risk of subsequent overt stroke [10–12].

#### Risk of cognitive decline

Cognitive deficits have been demonstrated in patients with SCD using validated tests for general intelligence, visual processing and academic achievement. Several studies have reported poorer global intellectual function in patients with SCIs [12, 22, 26, 32, 38, 64, 69, 76, 77]. Of the nine included studies in which an association between lower cognitive test scores and SCIs was evaluated, such an association was found in eight. The CSSCD study showed that children with SCD and SCIs scored lower on full-scale intelligence quotient (IQ) (*p* < 0.003), verbal IQ (*p* < 0.01), reading (*p* < 0.04) and math achievement tests (*p* < 0.04), than children with normal MRI findings [12, 76].

Although overt stroke is an obvious cause of neurologic abnormality and cognitive impairment [12], cognitive deficits also occur in patients without evidence of focal brain injury [53, 69, 78–80]. The CSSCD study found that children with a persistently normal cerebral MRI during the entire 10-year study period still presented with a decline of 1.5 IQ points per year [76]. In addition, Hogan et al. showed that lower intellectual functioning in children with SCD is partly explained by chronic hypoxia due to severe anaemia. This suggests that patients with normal MRI results may also have a constrained intellectual development [81]. Other studies conformingly show that low haemoglobin levels are a stronger predictor of neurocognitive function than SCIs [53, 69, 78].

#### Risk of recurrent and progressive SCIs or overt stroke

SCIs are associated with an increased risk of subsequent stroke in patients with SCD. The CSSCD study was the first to report that the presence of SCIs is a risk factor for additional neurological injury, with a 14-fold increase in the risk of overt stroke, as 25% of children with SCIs presented with new or enlarged lesions at follow-up [10]. These findings have been confirmed in more recent publications, including in a study in which SCIs in very young children were associated with subsequent progressive ischaemia and a higher risk of overt stroke [59]. Further supportive of progressive ischaemia are the studies which report that many patients with SCIs present with more than one lesion [20, 21, 27, 28, 32, 33, 36, 50, 77].

## Discussion

### Challenges regarding reviewed studies

Detection of SCIs is dependent upon the sensitivity and specificity of cerebral MRI scans and the definition of the radiological appearance. Importantly, advances in MRI technology lead to major heterogeneity when studying the prevalence, incidence and risk factors for the occurrence of SCIs in SCD. However, despite advancing neuroimaging technologies and therefore possible enhanced detection of SCIs, we did not find a rise in SCI prevalence in included studies over the years. MRI parameters varied widely in the studies included in this systematic review, with magnetic field strength ranging from 0.6 to 7.0 T and slice thickness ranging from 1.0 to 6.0 mm. One study used 7-T MRI and identified many more intracerebral lesions when compared to 3-T MRI scanning, both in patients and in controls, with SCI prevalence rates as high as 90% and 70%, respectively [51]. Although this study was excluded from our analysis as an outlier due to the extreme *Z*-score in order to not distort the overall analysis, it does suggest that SCI prevalence rates may actually be much higher when patients are screened with high-field magnet strength MRI. However, in accordance with the variability in SCI definitions utilized in the literature, the same study also observed that almost all lesions were smaller than 5 mm, with a majority even smaller than 3 mm [51]. When so small, lesions may be easily confused with dilated perivascular spaces, also known as Virchow-Robin spaces, which may lead to an overall overestimation in prevalence [82].

Most studies did not define whether the performed MRI scans were reviewed with knowledge of patients’ medical histories and whether or not neurological examination was performed by a neurologist. Because infarcts are classified as silent, if they—by definition—lack stroke-like symptoms, a haematologist may miss subtle neurological anomalies that may classify as a deficit in neurological examination by a neurologist.

We excluded studies with patients who had a history of overt stroke to ensure that no radiological apparent stroke was mistaken as SCIs. However, as SCIs have been identified as a risk factor for progression of SCIs and development of overt stroke, a large portion of stroke patients will also have additional SCIs. This was not taken into consideration in our analyses, and therefore, this exclusion criterion may have led to an underestimation in the prevalence of SCIs.

Most studies consisted of a heterogeneous group of patients. A few studies included individual patients on varying treatment regimens such as hydroxyurea medication or chronic red cell blood transfusions. Although currently there is no high-level evidence supporting these therapies as preventive for the development of SCIs [7], estimations of SCI prevalence may have been influenced by specific treatment regimens.

Finally, the absence of robust findings regarding the risk factors for the occurrence of SCIs is most probably due to the small patient sample sizes as well as weak associations. Tabulating the results in our systematic review was challenging due to the differences in definitions, heterogeneity of patients and treatment regimens and varying measurement methods and statistical analyses of many of the risk factors. Few studies explicitly stated the variables that were adjusted for in the multivariable analyses. In addition, the extent to which vascular risk factors are actually dependent upon one another is debatable, and interactions between the risk factors may have been overlooked by used analytic methods.

### Future directions for research

The study limitations mentioned above provide several considerations for future studies. In particular, the use of a consistent definition of SCIs is crucial. We suggest a minimal MRI field strength of 1.5 T with 3-mm-thick slices or thinner and at minimum the inclusion of FLAIR sequences. More specifically, lesions less than 3 mm in size should be excluded to minimize misdiagnosis of SCIs. In addition, reviewing of MRI scans as well as patient examination should be performed by experienced specialists.

Importantly, most studies were performed in children with SCD and limited attention has been given to adult patients. Longitudinal studies in patients over 16 years of age are necessary to understand the natural course of SCIs and their clinical relevance in adults with SCD. When designing future studies, it is essential to include matched control groups of healthy individuals, as siblings of patients with SCD often have sickle cell trait which is reported to possibly also impact cognition [48, 78, 83, 84]. Moreover, it is important to realize that healthy individuals also accumulate white matter hyperintensities with increasing age, which do not necessarily reflect SCIs. Epidemiological estimates have shown that the prevalence of SCIs detected by MRI screening is between 10 and 20% in the general population, with a strong association between SCI prevalence and age of the population assessed [82]. Unfortunately, the prevalence and incidence data on SCIs in populations younger than 45 years are lacking. Appreciation for possible SCIs in healthy children and young adults is essential to better understand SCI implications in SCD.

Lastly, further research is needed to determine the risk factors for and mechanisms of cognitive impairment in SCD in the absence of overt infarcts and SCIs. To this end, it is essential to differentiate between disease-related effects on brain function, indirect effects of chronic illnesses and psychosocial and socioeconomic factors [80]. This requires both longitudinal quantitative MRI and neuropsychological studies in combination with demographic and clinical variables.

## Conclusions

SCIs are common in patients with HbSS and HbSβ^0^ SCD with a weighted prevalence of 29.5%. SCIs occur more often in patients with HbSS and HbSβ^0^ SCD when compared to other SCD genotypes and healthy controls, as respectively SCIs were found in 9.2% of HbSC and HbSβ^+^ patients and in 9.8% of controls.

Although the prevalence estimates varied widely across studies, data from this systematic review show a significant association between increasing patient age and SCI prevalence, which is consistent with an effect of age on cerebrovascular disease in SCD. Risk factor analyses showed no clear association between prevalence of SCIs and studied risk factors. Additional neuroimaging of patient populations with TCD, MRA or ASL may elucidate the pathogenesis and risk factors for the development of SCIs in SCD long term. Larger, prospective and controlled clinical, neuropsychological and neuroimaging studies are needed to understand how SCD and SCIs negatively affect cognition. Such studies may also provide a starting point for the identification of potential targets for preventive therapies by a better understanding of underlying pathophysiological mechanisms.

## Supplementary Information

**Additional file 1.** Systematic search.

**Additional file 2: Table 1.** Prevalence of silent cerebral infarcts in control subjects.

## Data Availability

The datasets used and/or analysed during the current study are available from the corresponding author on reasonable request.

## Abbreviations

ASL: Arterial spin labelling
CSSCD: Cooperative Study of Sickle Cell Disease
CT: Computed tomography
FLAIR: Fluid-attenuated inversion recovery
MRA: Magnetic resonance angiography
MRI: Magnetic resonance imaging
SCD: Sickle cell disease
SCIs: Silent cerebral infarctions
SIT trial: Silent Cerebral Infarct Transfusion trial
STOP trial: Stroke Prevention trial
TCD: Transcranial Doppler
