# Supplementary material for: Silent cerebral infarcts in patients with sickle cell disease: a systematic review and meta-analysis
Source: BMC Med. 2020 Dec 22;18:393. doi: 10.1186/s12916-020-01864-8 (PMC7754589; doi:10.1186/s12916-020-01864-8)
Supplement: Supplementary file 1 — Additional file 1. Systematic search. [file 12916_2020_1864_MOESM1_ESM.docx]

**Additional file 1 - Systematic search**

**Embase**
('sickle cell anemia'/exp OR 'sickle cell'/de OR ('sickle cell*'):ab,ti) AND ('nuclear magnetic resonance imaging'/exp OR ((magnetic* NEAR/3 resonan*) OR mri OR fmri):ab,ti) AND ('brain infarction'/exp OR 'brain ischemia'/exp OR (((brain OR silent OR cerebr*) NEAR/3 (infarct* OR ischem* OR ischaem*)) OR ((brain OR cerebr* OR stroke OR cva OR ischemi* OR ischaemi*) NEAR/3 silent*)):ab,ti) NOT ([animals]/lim NOT [humans]/lim) NOT ([Conference Abstract]/lim OR [Letter]/lim OR [Note]/lim OR [Editorial]/lim) AND [english]/lim

**MEDLINE**
(Anemia, Sickle Cell/ OR sickle cell/ OR (sickle cell*).ab,ti.) AND (Magnetic Resonance Imaging/ OR ((magnetic* ADJ3 resonan*) OR mri OR fmri).ab,ti.) AND (brain infarction/ OR brain ischemia/ OR (((brain OR silent OR cerebr*) ADJ3 (infarct* OR ischem* OR ischaem*)) OR ((brain OR cerebr* OR stroke OR cva OR ischemi* OR ischaemi*) ADJ3 silent*)).ab,ti.) NOT (exp animals/ NOT humans/) NOT (letter* OR news OR comment* OR editorial* OR congres* OR abstract* OR book* OR chapter* OR dissertation abstract*).pt. AND english.la.

**Web of Science**
TS=((("sickle cell*")) AND ((neuroimag* OR (magnetic* NEAR/2 resonan*) OR mri OR fmri OR (compute* NEAR/2 tomogra*) OR ((ct OR cat) NEAR/1 scan*) OR ((brain OR cerebr* OR stroke OR cva OR ischemi* OR ischaemi* OR infarct*) NEAR/2 silent*))) AND ((((brain OR silent OR cerebr*) NEAR/2 (infarct* OR ischem* OR ischaem*)) OR ((brain OR cerebr* OR stroke OR cva OR ischemi* OR ischaemi*) NEAR/2 silent*))) )

**Cochrane Central**(('sickle cell*'):ab,ti) AND (((magnetic* NEAR/3 resonan*) OR mri OR fmri):ab,ti) AND ((((brain OR silent OR cerebr*) NEAR/3 (infarct* OR ischem* OR ischaem*)) OR ((brain OR cerebr* OR stroke OR cva OR ischemi* OR ischaemi*) NEAR/3 silent*)):ab,ti)

**Google Scholar**"sickle cell" mri "silent brain|cerebral|stroke|cva|ischemia|ischaemia|ischemic|ischaemic|infarct|infarction"
