# Supplementary material for: Silent cerebral infarcts in patients with sickle cell disease: a systematic review and meta-analysis
Source: BMC Med. 2020 Dec 22;18:393. doi: 10.1186/s12916-020-01864-8 (PMC7754589; doi:10.1186/s12916-020-01864-8)
Supplement: Supplementary file 2 — Additional file 2: Table 1. Prevalence of silent cerebral infarcts in control subjects. [file 12916_2020_1864_MOESM2_ESM.docx]

**Additional table 1**

**Additional table 1. Prevalence of silent cerebral infarcts in control subjects**

| Author | Sample  size | HbS carrier status | Mean age (years) | Prevalence SCIs in all controls % | Prevalence SCIs in HbAS controls % |
| --- | --- | --- | --- | --- | --- |
| Asbeutah et al. 2014 (42) | 26 | NA | 10.0 | 0 | NA |
| Baldeweg et al. 2006 (36) | 31 | NA | 15.7 | 0 | NA |
| Coloigner et al. 2017 (37) | 21 | 9 HbAS, 12 HbAA | 22.3 | 9.5 | Unspecified |
| Dowling et al. 2012 (44) | 30 | NA | 9.8 | 26.7 | NA |
| Ford et al. 2017 (40) | 22 | 11 HbAS, 11 HbAA | NA | 27.3 | HbAS: 45.5, HbAA: 9.1% |
| Issar et al. 2018 (45) | 74 | 18 HbAS, 56 HbAA | NA | 0 | NA |
| Oguz et al. 2003 (46) | 7 | NA | 11.0 | 0 | NA |
| Václavů et al. 2019 (39) | 11 | 2 HbAS, 9 HbAA | 37.4 | 45.5 | Unspecified |
| Vichinsky et al. 2010 (47) | 44 | 44 HbAA | 33.1 | 11.4 | NA |

*Abbreviations; SCIs: silent cerebral infarctions, NA: not available.*
